# Novel use of an adjustable single 8–0 polypropylene suture of scleral fixation without conjunctival dissection

**DOI:** 10.1186/s12886-020-01558-y

**Published:** 2020-07-25

**Authors:** Bin Mo, Song-Feng Li

**Affiliations:** Beijing Tongren Eye Center, Beijing Ophthalmology & Visual Sciences Key Laboratory, Beijing Tongren Hospital, Capital Medical University, No. 1 Dong Jiao Min Xiang, Eastern District, Beijing, 100730 China

**Keywords:** Transscleral suture fixation, Adjustable, 8–0 polypropylene suture, Without conjunctival dissection

## Abstract

**Background:**

This report serves to describe the use of a novel adjustable single 8–0 polypropylene suture for scleral fixation without conjunctival dissection, and to describe related clinical outcomes associated with this approach.

**Methods:**

In this study, we retrospectively reviewed 28 eyes from 27 patients that underwent scleral fixation of the intraocular lens (IOL) without conjunctival dissection using an adjustable single 8–0 polypropylene suture at the Beijing Tongren Eye Center between April 2018 and April 2019. For this surgical approach, a 23-gauge infusion cannula was set, after which two Hoffmann scleral pockets were created. Next, 8–0 polypropylene sutures were inserted into the eye guided by 10–0 polypropylene sutures of a long straight needle. The 8–0 suture was then used to fix the haptic IOs. Finally, these 8–0 polypropylene sutures were removed from the scleral pockets, and knots were tightened with the adjustable single suture. Primary outcomes included visual acuity and postoperative complication incidence.

**Results:**

For this study, outcomes for 28 eyes from 27 patients (9 female, 18 male) were assessed. Patients had a mean age of 54 ± 15.11 years-old and were followed for an average of 10.18 ± 2.76 months postoperatively. Uncorrected visual acuity in these patients improved significantly from a preoperative value of 1.269 ± 0.464 logMAR to a 3-month postoperative value of 0.409 ± 0.413 logMAR (*p* = 0.000). The majority of postoperative complications in these patients were temporary and self-limiting, including corneal edema (35.71%), hypotony (14.29%), elevated intraocular pressure (28.58%), and mild hyphema (7.14%). No evidence of exposure or erosion of the trimmed suture end was detected in any patients. An ultrasound biomicroscope was able to readily detect the IOL and all sutures, and IOLs were found to be well-centered without any dislocation, tilting, or subluxation upon follow-up.

**Conclusions:**

An adjustable single 8–0 polypropylene suture can reliably and effectively be used for scleral fixation without conjunctival dissection for the treatment of patients with aphakia or inadequate posterior capsule support. The novel procedure described herein may therefore be an effective means of minimizing the risk of suture-related complications in patients undergoing scleral-fixated IOL implantation.

**Trial registration:**

Retrospective case series study, not applicable. NCT04476264.

## Background

A number of surgical approaches have been employed for intraocular lens (IOL) implantation in eyes lacking posterior capsular support, such as anterior chamber IOL, iris-fixated IOL, sulcus-fixated IOL, and scleral-fixated IOL implantation via suture-based or suture-free intrascleral fixation [1]. Anterior chamber IOL implantation, however, is rarely conducted as it is associated with relatively high rates of complications such as glaucoma, limited pupillary mobility, chronic inflammation, and corneal decompensation.

Transscleral suture-mediated IOL fixation was first described by Malbran in 1986 [2], and is a relatively common surgical approach owing to its low incidence of postoperative complications. Prior studies have described certain suture-related complications associated with this approach, including inflammation, suture erosion, and suture degradation [3, 4]. Efforts to prevent these complications have employed approached including the rotating of suture knots into the eye and covering suture ends with scleral pockets or flaps [5–9].

A key concern associated with this procedure is the appropriate selection of suture materials and suture knotting approaches. Utilizing 10–0 polypropylene sutures has become increasingly unpopular, as they tend to degrade over time, ultimately resulting in IOL subluxation and dislocation. More recently, researchers have utilized 8–0 polypropylene sutures as an alternative owing to their high tensile strength and comparatively lower susceptibility to degradation [10].

The present study serves to describe a novel approach to utilizing a single adjustable 8–0 polypropylene suture for scleral fixation without conjunctival dissection and to evaluate safety and efficacy outcomes associated with this technique.

## Methods

### Patients

Surgical outcomes from 28 eyes from 27 patients that underwent treatment using a single adjustable 8–0 polypropylene suture to achieve IOL scleral fixation without conjunctival dissection at the Beijing Tongren Eye Center between April 2018 and April 2019 were retrospectively reviewed. All operations were conducted by a single experienced surgeon (S-F L). This study was consistent with the Declaration of Helsinki.

Data from patients with a < 6-month postoperative follow-up or with incomplete operative or postoperative medical records were excluded from this study. Enrolled patients had complete records pertaining to their visual acuity (VA), slit-lamp photographs, and ultrasound biomicroscope (UBM) findings.

During their initial baseline visit, each patient was subjected to comprehensive ophthalmic evaluations of uncorrected and best-corrected visual acuity (BCVA), as well as intraocular pressure, slit-lamp biomicroscopy, indirect ophthalmoscopy, color fundus photography analyses. Postoperative uncorrected VA was measured at 3 months postoperatively with a Snellen chart and was converted to the logarithm of the minimum angle of resolution (logMAR) VA for computational purposes. The following LogMAR cutoffs were used for non-numeric VA [11]: able to count fingers = 1.7 LogMAR; able to detect hand movement = 2.0 LogMAR; light perception = 2.3 LogMAR; no light perception = 3.0 LogMAR.

### Surgical techniques

Initially, a standard 23-gauge transconjunctival trocar was inserted at 3.5 mm from the limbus at the inferotemporal quadrant, and intraocular pressure was maintained via infusion. When necessary, two additional 23-gauge trocars were inserted for combined pars plana vitrectomy.

**Fig. 1:**
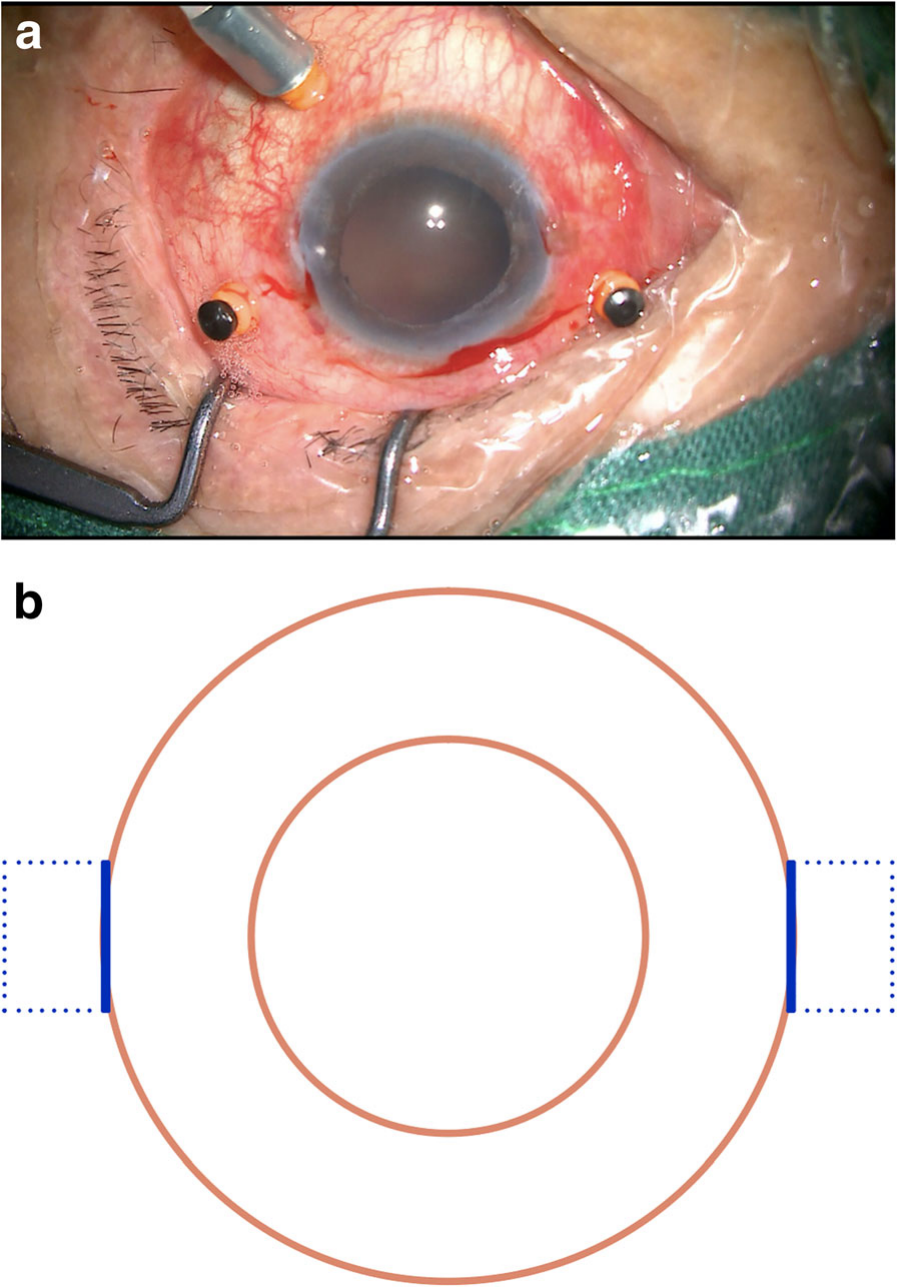
Two scleral pockets without conjunctival dissection were created posteriorly from the two opposing incisions. **a**. A photo taken during surgery. **b**. A schematic of the modified procedure

A sharp #11 blade and a 1.25 mm mini-crescent blade were then used to create 1/3 corneal thickness incisions to generate two opposing 3 × 3 mm Hoffmann scleral pockets [8] (Fig. 1a-b).

**Fig. 2:**
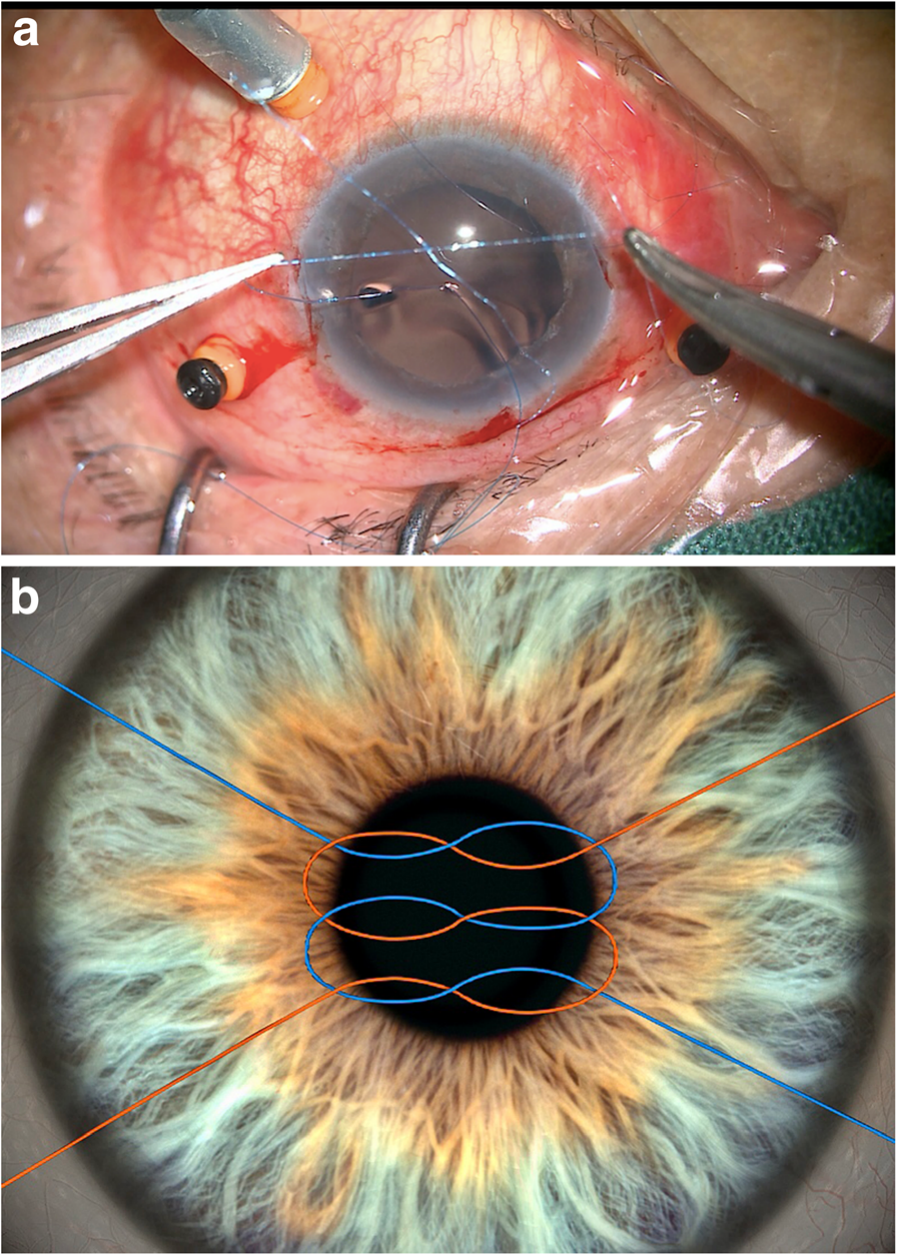
An 8–0 polypropylene suture was knotted with a 10–0 polypropylene suture. **a**. A photo taken during surgery. **b**. A schematic of the modified knotting procedure

**Fig. 3:**
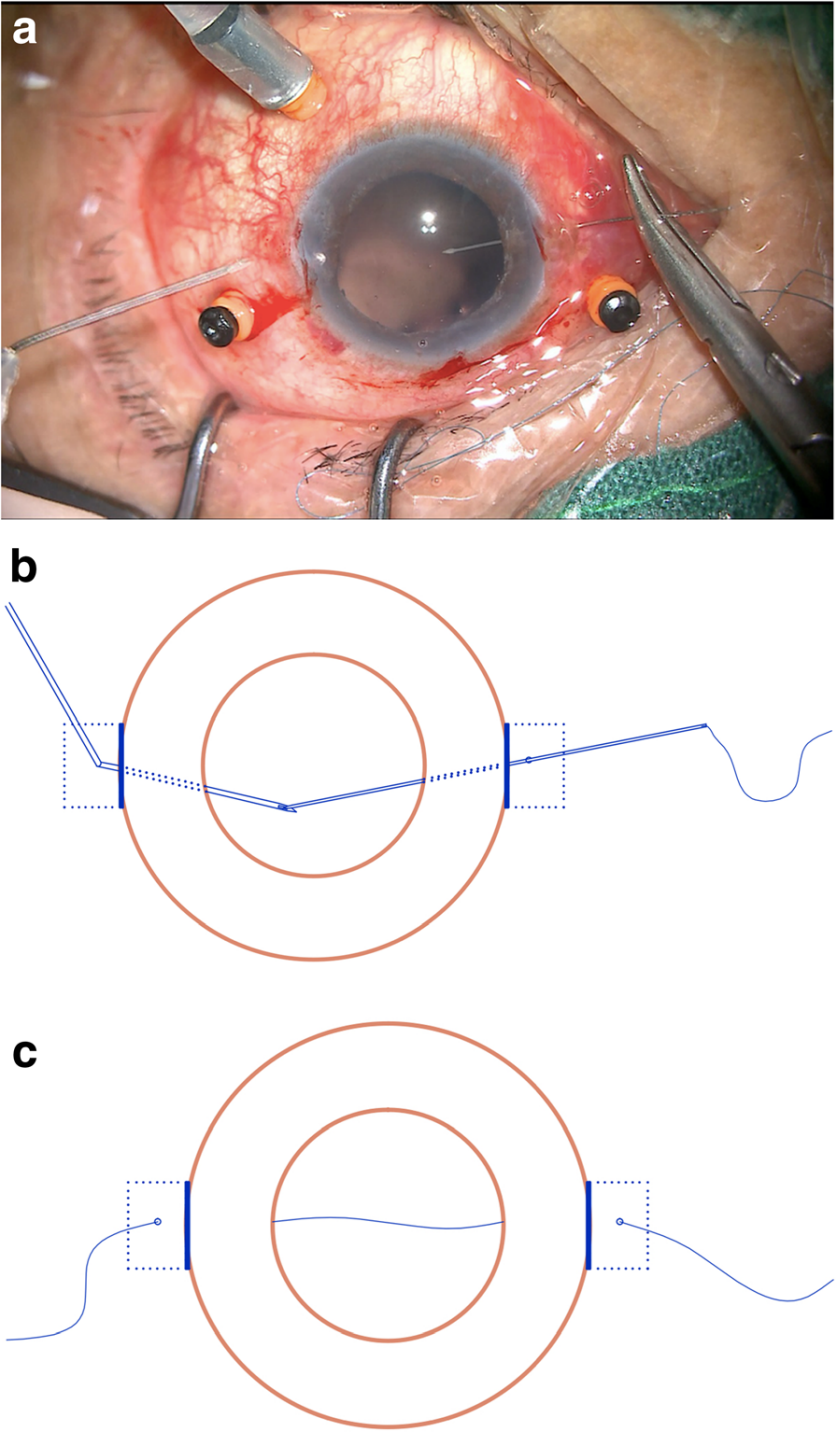
An overview of the insertion of an 8–0 polypropylene suture into the eye. **a**. Surgical photos demonstrating the presence of a 10–0 polypropylene suture with an 8–0 polypropylene suture of a long straight needle that was inserted into the eye on one side via passing through the conjunctiva and the full thickness of the scleral pocket at 1.5 mm posterior to the surgical limbus. **b**. A schematic of the procedure wherein the long straight needle was docked with a 27-gauge needle and externalized. **c**. A schematic of the procedure whereby an 8–0 polypropylene suture was inserted into the eye

A curved 8–0 polypropylene suture (W2777, PROLENE, Ethicon) was prepared and knotted with a 10–0 polypropylene suture of a long straight needle (W1713, PROLENE, Ethicon) (Fig. 2a-b). A 27-gauge needle was then passed through the conjunctiva and the Hoffmann scleral pocket at 1.5 mm posterior to the surgical limbus to generate a scleral inlet, after which the long straight needle 10–0 polypropylene suture with the 8–0 polypropylene suture was inserted into the eye via this inlet (Fig. 3a). Next, another 27-gauge needle was used to penetrate the eye on the opposite side via the conjunctiva and the scleral pocket, after which the long straight needle penetrated the lumen of the 27-gauge needle and was externalized from the eye (Fig. 3b-c). The 10–0 polypropylene suture was then cut away from the 8–0 suture, and a 3.0 mm clear corneal incision was made at the superior position, after which a chopper was used to pull the 8–0 suture out via the superior corneal incision, after which it was cut at the center. The 8–0 suture ends were then tied symmetrically to the closed-loop haptics of the IOL, which was then inserted into the posterior chamber. The sutures were then pulled to center the optics, and severe iris and pupil damage was repaired.

**Fig. 4:**
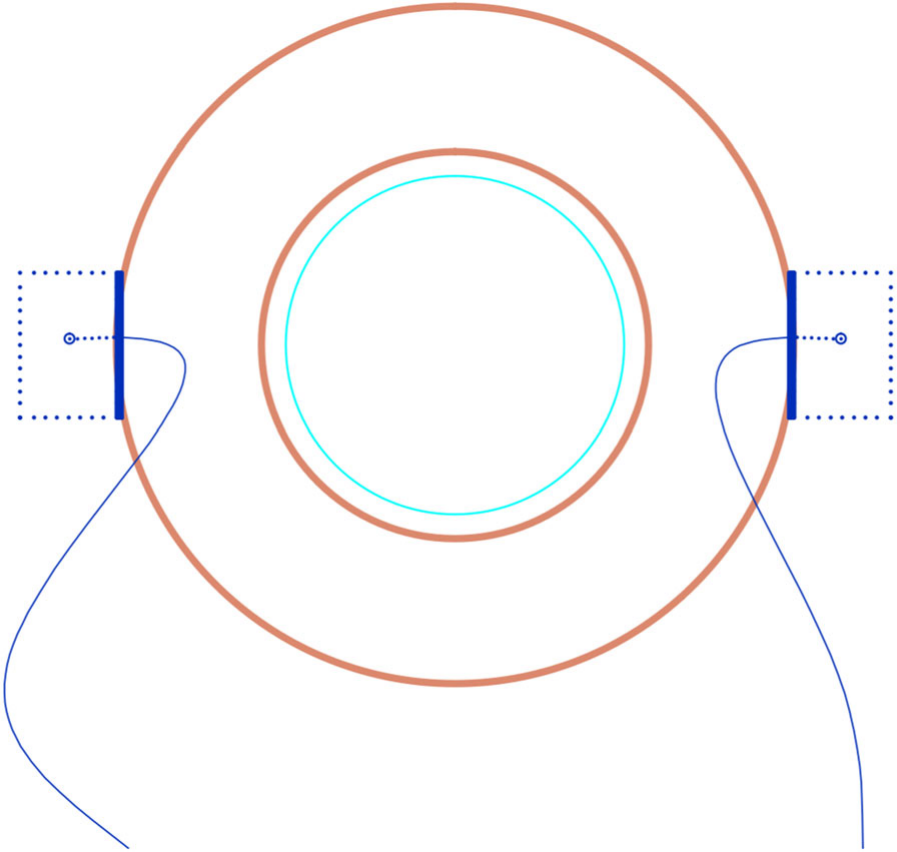
A schematic of the steps involved in suture retrieval from the scleral pockets

**Fig. 5:**
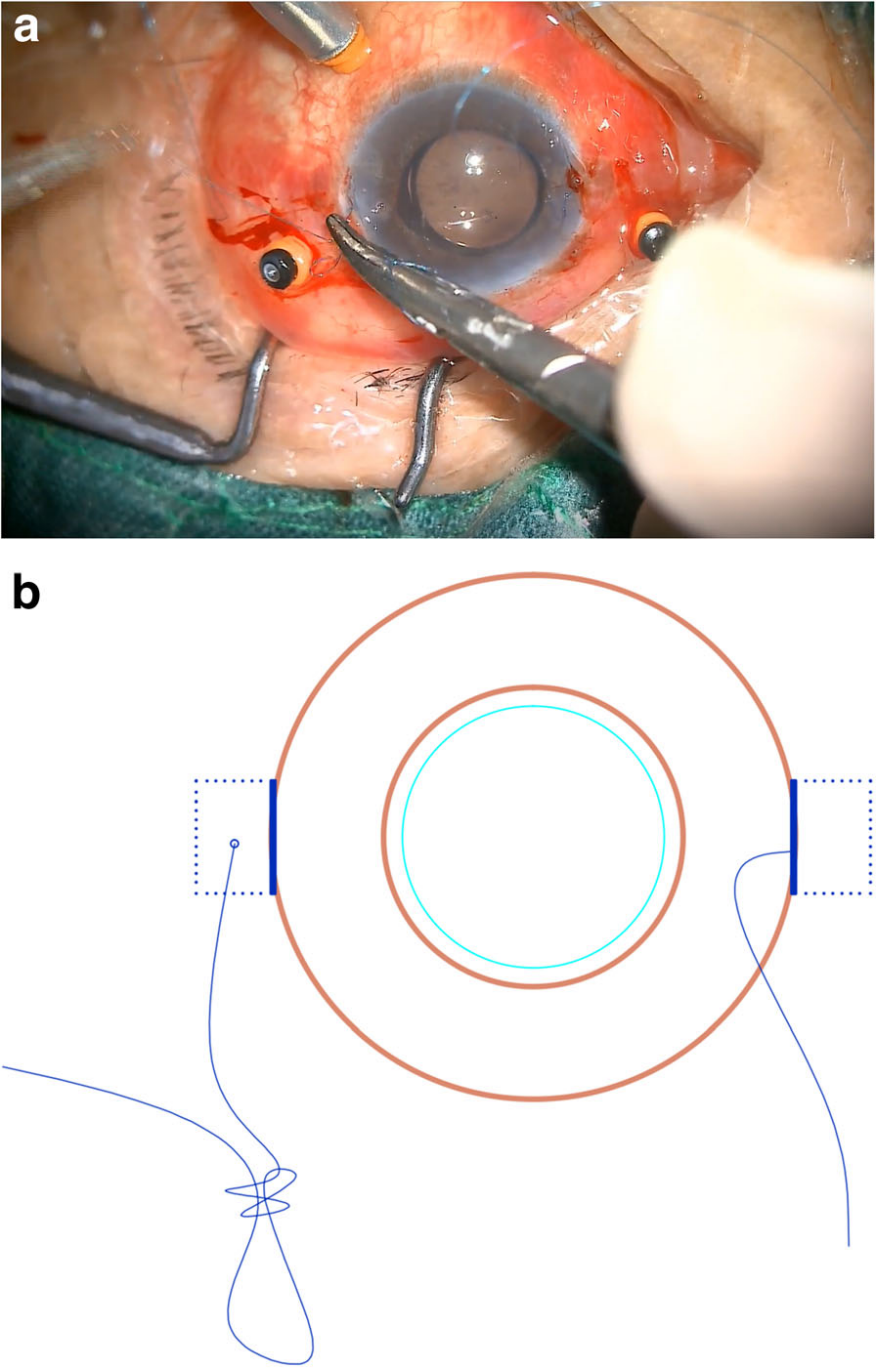
The free end of the suture was wrapped two times, and the proximal end was grasped on both ends in order to tie the suture. **a**. A photo taken during surgery. **b**. A schematic of the modified procedure

**Fig. 6:**
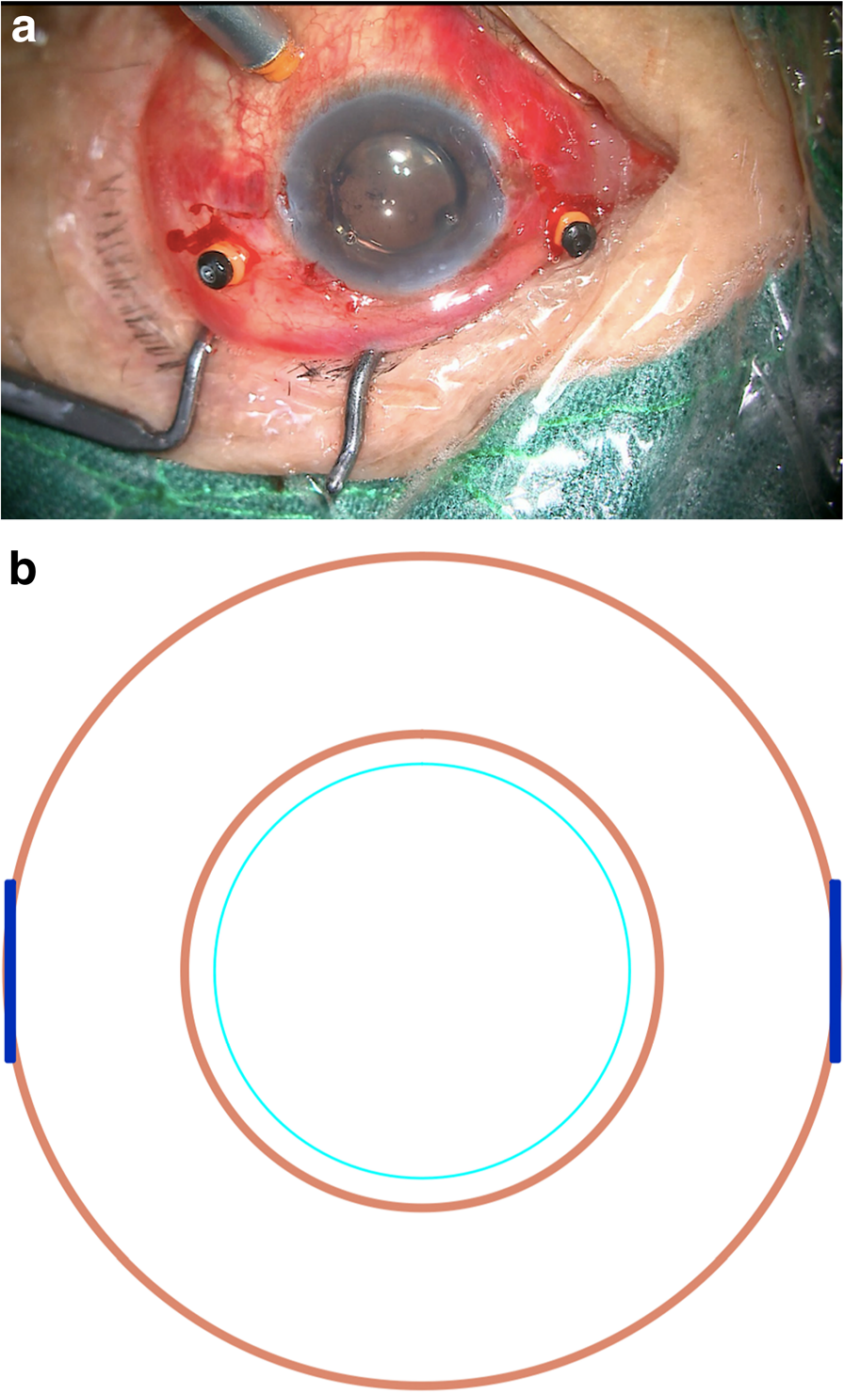
The IOL was well centered, with suture ends lying flat within the prepared scleral pockets. **a**. A photo taken during surgery. **b**. A schematic of the modified procedure

A chopper was then used to extract the 8–0 sutures from the Hoffmann scleral pockets (Fig. 4), after which the free suture ends were wrapped twice and grasped at the proximal end to tie the knot (Fig. 5a-b). The tightness of the proximal end of the suture was then adjusted to properly center the IOL, and the knot was fixed by an adjustable single suture. The suture ends remained ~ 4 mm long and were buried under the scleral pocket (Fig. 6a-b).

This same approach can additionally be performed for the opposite haptic using the corresponding opposing scleral pocket. Then, 10–0 nylon was used to suture the superior clear corneal incision, after which the 23-gauge trocars were removed and suturing of the remaining incision was performed. After suturing was complete, all patients received a subconjunctival injection of dexamethasone (2.5 mg).

### Statistical analysis

SPSS v22.0 (SPSS Inc., IL, USA) was used to analyze all visual acuity data, which were reported as means±SD. Data were compared via pairwise Student’s t-test, with *p* < 0.05 as the significance threshold.

## Results

### Basic patient characteristics

Patient baseline characteristics including their ophthalmic history, surgical indications, and lens status are compiled in Table 1. This study enrolled 28 total eyes from 27 patients (9 female, 18 male). These patients had a mean age of 54 ± 15.11 years old and were followed for an average of 10.18 ± 2.76 months postoperatively. Causes of inadequate capsular support in these patients included blunt or penetrating ocular trauma, previous complicated cataract surgery, or spontaneous crystalline lens subluxation.

**Table 1:** Basic patient characteristics

Total number of eyes	28
Total number of patients	27
Male:Female	18:9
Mean age (y)	54 ± 15.11
Follow-up time (m)	10.18 ± 2.76
Cause of lens complications, n (%)	
Ocular trauma	11 (39.29%)
Penetrating ocular trauma	3
Blunt ocular trauma	8
Cataract surgery complications	8 (28.57%)
Spontaneous subluxation of the crystalline lens	9 (32.14%)

**Table 2:** Postoperative complications

Postoperative complications	Number of eyes (%)
Transient corneal edema	10 (35.71%)
Temporary hypotony	4 (14.29%)
Temporary intraocular pressure elevation	8 (28.58%)
Mild hyphema	2 (7.14%)
Other postoperative complications	0

### Surgical procedures and post-operative uncorrected VA

The key facet of this surgical approach was the use of a single adjustable 8–0 polypropylene suture for the scleral fixation of the IOL without conjunctival dissection. We therefore achieved IOL fixation via a single-pass approach with these adjustable 8–0 polypropylene sutures. Two different varieties of closed-loop foldable one-piece IOLs were utilized in the present study. Uncorrected VA improved from a preoperative mean of 1.269 ± 0.464 logMAR to a significantly higher mean 3-month postoperative value of 0.409 ± 0.413 logMAR (T-test, *p* = 0.000).

### IOL and tunnel location

**Fig. 7:**
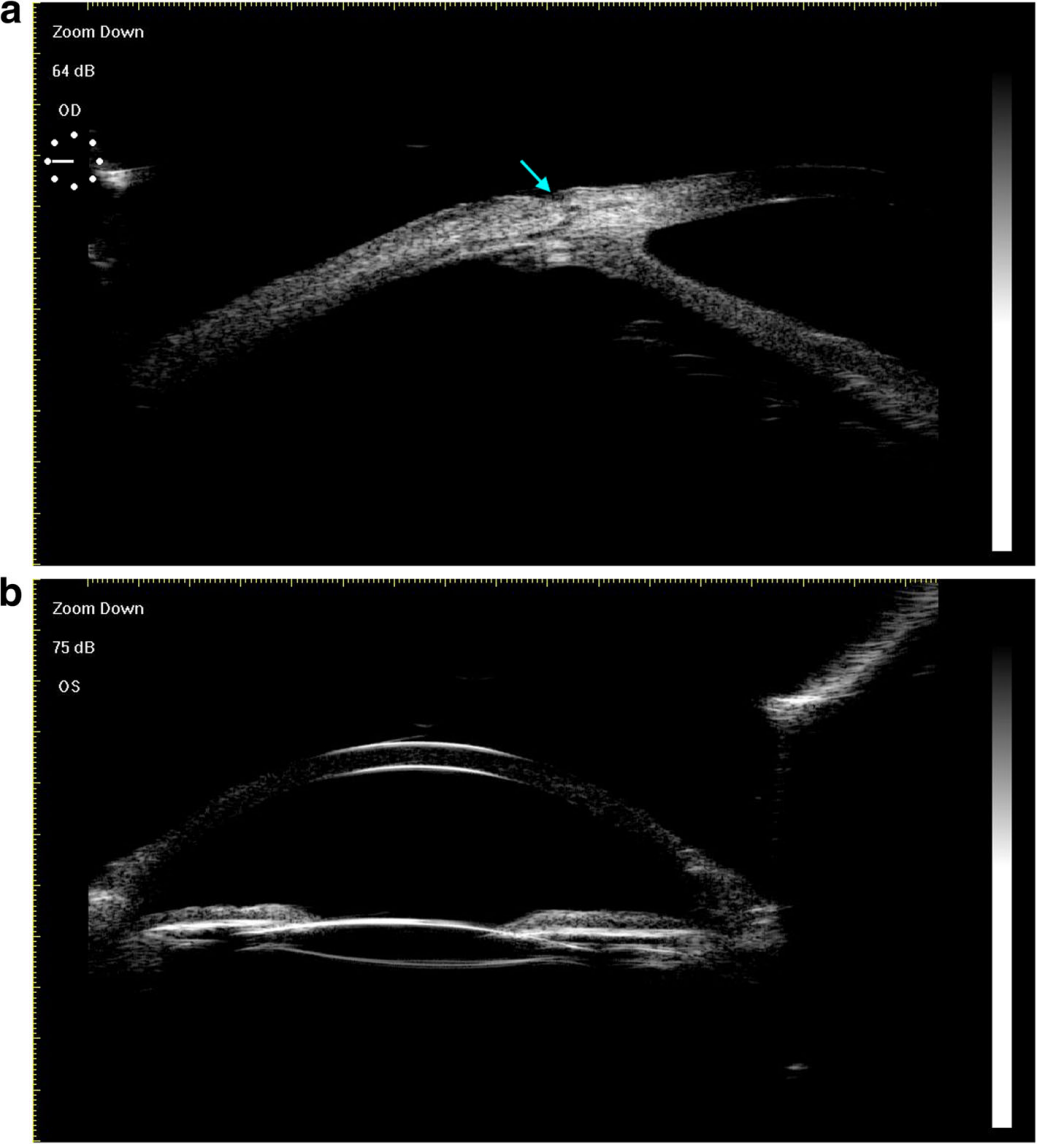
UBM examination. **a**. Tunnels containing the sutures were clearly detected by UBM at two months postoperatively (blue arrow). **b**. IOL centration was readily detectable via UBM

IOL tunnels containing sutures were clearly visible by UBM at two months postoperatively (Fig. 7a). We additionally utilized slit-lamp photography and UBM to assess whether or not the IOL was centered in each eye, and in all cases we found that IOLs were well centered without evidence of subluxation, tilt, or dislocation (Fig. 7b).

### Postoperative complications

Two eyes in the present study exhibited transient minor ciliary bleeding that did not require hemostasis during ciliary sulcus penetration, while no other intraoperative complications were observed. Postoperative complications section, line 6, Two eyes in the present study exhibited transient minor ciliary bleeding that did not require hemostasis during ciliary sulcus penetration, while no other intraoperative complications were observed. Postoperative complications in the present patient cohort included corneal edema in 10/28 eyes (35.71%), hypotony (6–10 mmHg) in 4/28 eyes (14.29%), and temporary elevation of intraocular pressure (>21 mmHg) in 8/48 eyes (28.58%) (Table 2). These complications resolved spontaneously within a four-week period and no evidence of exposure or erosion of the trimmed suture ends was observed. In addition, no major postoperative complications such as vitreous hemorrhage, corneal endothelial decompensation, persistent uveitis, suprachoroidal hemorrhage, cystoid macular edema, retinal detachment, or endophthalmitis were observed.

## Discussion

Scleral fixated posterior chamber (PC) IOL implantation was first discussed in 1986 [1], and focuses on an approach that preserves anterior chamber integrity, minimizes uveal contact, and avoids disrupting the ocular anatomy. Outcomes of this approach are unrelated to iris tissue presence. However, complications of this approach when it is conducted in concert with suturing techniques include suture-induced erosion, suture exposure, and suture rupture-related IOL dislocation. Optimal suture selection has been suggested as a viable approach to improving patient outcomes associated with this approach, and suture breakage rates have been reported to range from 1.3–27.9% after 3–8 years when 10–0 polypropylene sutures are used [12]. Owing to their stability, stress resistance, and biocompatibility, polypropylene sutures have long been applied in a range of ophthalmic surgeries [13]. Work by Stewart and Khan suggests that the use of 9–0 or 8–0 polypropylene sutures can minimize or eliminate the risks of complications associated with 10–0 sutures such as suture degradation and consequent IOL malposition, making such sutures better suited to IOL fixation [14–16]. This has led some surgeons to adopt the use of 9–0 or 8–0 polypropylene sutures for such operations owing to their greater tensile strength and diameter. However, the instructions for polypropylene sutures clearly indicate that polypropylene sutures can neither be absorbed nor degraded or weakened by tissue enzymes. We therefore speculate that suture-induced erosion is not the result of polypropylene suture degradation, and is instead a consequence of the cutting effect of this suture on the IOL and surrounding tissues. Thinner sutures may have a stronger cutting effect, and as such, we elected to utilize 8–0 polypropylene sutures in the present study to minimize these cutting effects.

We utilized 8–0 polypropylene sutures for PC IOL fixation, and at present only curved needles are available for such sutures, preventing them from being directly inserted into the eye. We therefore overcame this limitation by inserting the 8–0 sutures into the eye using 10–0 polypropylene sutures with a long straight needle for guidance. The curved 8–0 suture needle was first cut, and then the 8–0 and 10–0 suture were knotted together. The single-arm 10–0 suture was then inserted into the eye with the long straight needle, which penetrated the lumen of the 27-gauge needle and was subsequently externalized from the eye. John et al. have previously detailed a four-point approach to PC IOL scleral fixation with 8–0 polypropylene sutures by first passing free suture ends through the corneal incision and out of the sclerotomy sites with 25-gauge forceps [10]. In contrast, we used another method to insert the 8–0 polypropylene suture into the eye. We inserted the 8–0 polypropylene suture into a 1 ml (25G) syringe needle that was subsequently inserted into the eye. These approaches, however, are complex and have the potential to cause significant wound leakage. This 1 mL syringe needle is also not sufficiently long to achieve eyeball penetration in those patients that have a longer eye axis. The simplified method that we have described in the present study is far easier to learn and overcomes the potential limitations and risks of repeatedly passing a long needle through the globe while minimizing the risk of wound leakage. We observed minor temporary ciliary bleeding during ciliary sulcus penetration in two eyes in the present study when using this approach, and there were no instances of suture knot dragging during penetration. This procedure will be further simplified in the future by the production of 8–0 polypropylene sutures with straight needles.

The fixation of the PC IOL into the ciliary sulcus remains a significant challenge. Herein, we achieved such fixation via a novel single-pass approach with an adjustable single suture. The majority of surgical approaches in this context employ conjunctival peritomy and necessitate the generation of a scleral flap or glove for IOL haptic or suture embedding. In contrast, Hoffmann et al. proposed the generation of a “reverse” scleral pocket that was created from a clear corneal incision without conjunctival dissection as a means of fixing in-the-bag IOL dislocation [8]. This approach has also been reported as a means of dealing with several IOL complications [7, 17]. There are a number of advantages to this scleral pocket approach as a means of achieving scleral fixation. For one, as these pockets originate from a clear corneal incision, they do not damage the conjunctiva or scleral surface and do not require conjunctival suturing, thereby preventing the scarring of these surfaces. This approach additionally reduced patient discomfort, postoperative astigmatism, and risk of postoperative suture exposure. Importantly, this technique also proves a good operative space for future surgical procedures for the treatment of glaucoma or other ocular conditions. Herein, we utilized a modified version of this approach to fixing IOLs in the ciliary sulcus. We generated Hoffmann scleral pockets and then inserted the 8–0 polypropylene suture into the eye using a 10–0 polypropylene suture with a long straight needle for guidance. A chopper was then used to pull these 8–0 sutures out of these two Hoffmann scleral pockets, and knots were tied and fixed with a single adjustable suture similar to that used for the treatment of glaucoma. This step is novel, and is also important for the success of the procedure. In their studies, Hoffmann and Yeung described approaches that utilized a double-pass technique to tie the sutures, but such an approach is associated with a higher risk of adverse events such as wound leakage and bleeding, in addition to requiring more steps and a greater amount of time [7, 8]. In order to decrease the potential for such complications, we modified this approach by passing a single suture that was then tied to itself. The free suture end was wrapped twice, with the proximal ends being grasped for typing, after which the IOL was centered via the tightening of the proximal suture ends. These ends were ~ 4 mm long, and were ultimately buried within the scleral pocket. We conducted classical two-point scleral fixation of a PC IOL in the ciliary sulcus, allowing us to adjust the IOL position by ensuring that the sutures were symmetrically positions and that the proximal end of these sutures was moderately tightened.

Herein, we utilized slit-lamp imaging and UBM to assess whether or not the IOLs were centered in these patients, revealing that all IOLs were well-centered without evidence of tilting, dislocation, or subluxation during follow-up. Aside from two cases of minor ciliary bleeding, no other intraoperative complications were observed among patients in the present study. Postoperatively, corneal edema occurred in 10/28 eyes (35.71%), hypotony (6–10 mmHg) in 4/28 eyes (14.29%), and elevated intraocular pressure (> 21 mmHg) in 8/48 eyes (28.58%). These complications all resolved within a four-week period, and we did not observe any exposure or erosion of the trimmed suture ends. Importantly, no serious postoperative complications such as vitreous hemorrhage, corneal endothelial decompensation, persistent uveitis, suprachoroidal hemorrhage, cystoid macular edema, retinal detachment, or endophthalmitis were detected.

The technique proposed herein is a safe and effective approach the reliably treating patients suffering from aphakia or with inadequate posterior capsule support. There are a number of advantages to this approach. For one, 8–0 polypropylene sutures have a larger diameter and greater tensile strength, thus reducing the cutting effect and enabling them to remain more stable within the pupillary area and the sclerotomy sites. Secondly, these 8–0 sutures were inserted into the eye using a single-armed 10–0 polypropylene suture of a long straight needle for guidance, which is beneficial given that this approach can readily be learned and can decrease the odds of leakage from the wound site. Third, the knots involved in this approach were tied and fixed using a single adjustable suture similar to those used to treat glaucoma via a single-pass approach with the potential to reduce the incidence of bleeding or wound leakage. This approach also necessitates less time and fewer steps than other interventional strategies. The use of a modified Hoffman scleral pocket can also reduce patient pain or discomfort, facilitating more rapid wound recovery while decreasing postoperative risks of suture exposure and astigmatism. Our approach has a wide range of potential applications for fixing various types of IOLs. Herein, we utilized two varieties of closed-loop foldable one-piece IOLs, but open-loop IOLs can similarly be used. In contrast, sutureless intrascleral fixation techniques are limited by scleral thickness, eye axis length, and IOL type [18–22], and are only compatible with three-piece IOLs with open-PMMA loop haptics. For this approach, it is also critical that the two IOL loops be fixed symmetrically between the intrascleral layers to reduce the rotation and decentration of the IOL, meaning that this operation is more technical and will require more time to master.

There are certain limitations to the present study. For one, this was a retrospective analysis with a relatively limited sample size. Secondly, we had a relatively limited 10.18 ± 2.76 month follow-up duration. We also did not estimate rates of late suture-associated complications for the 8–0 sutures, such as the cutting effect of these sutures over time and resultant instances of IOL malposition. Future studies enrolling more patients over a longer follow-up period will be essential in order to more fully resolve differences in long-term outcomes and complications associated with this operation, particularly among younger patients who will have the implanted IOL for a longer duration than most patients in the present study. Additional research comparing the safety and efficacy of this approach and other scleral fixation surgical methods is also warranted. It is also important to note that we did not specifically evaluate the degree of astigmatism in these patients, although we were able to estimate the degree to which the IOL was centered via UBM. In future studies, we will employ optometric approaches to directly clear the astigmatism of the IOL in treated patients.

In summary, the single-pass use of a single adjustable 8–0 polypropylene suture for scleral fixation without conjunctival dissection represents a novel, safe, effective, and reliable approach to treating patients suffering from aphakia or inadequate posterior capsule support. This treatment approach can achieve excellent IOL stability, and is associated with low rates of intraoperative and postoperative complications. Our procedure may therefore lower the risk of suture-related complications in patients undergoing scleral-fixated IOL implantation.

## Data Availability

The datasets used and/or analysed during the current study are available from the corresponding author on reasonable request.

## Abbreviations

AC: Anterior chamber
VA: Visual acuity
IOL: Intraocular lens
LogMAR: Logarithm of the minimum angle of resolution
PC: Posterior chamber
UBM: Ultrasound biomicroscope
